# Surgeons' Volume-Outcome Relationship for Lobectomies and Wedge Resections for Cancer Using Video-Assisted Thoracoscopic Techniques

**DOI:** 10.1155/2012/760292

**Published:** 2012-11-04

**Authors:** Guy David, Candace L. Gunnarsson, Matt Moore, John Howington, Daniel L. Miller, Michael A. Maddaus, Robert Joseph McKenna, Bryan F. Meyers, Scott J. Swanson

**Affiliations:** ^1^Associate Professor of Health Care Management, The Wharton School, University of Pennsylvania, 202 Colonial Penn Center, 3641 Locust Walk, Philadelphia, PA 19104, USA; ^2^S^2^ Statistical Solutions, Inc., 11176 Main Street, Cincinnati, OH 45241, USA; ^3^Healthcare Policy and Economics, Ethicon Endo-Surgery, 4545 Creek Road, Cincinnati, OH 45252, USA; ^4^Division of Thoracic Surgery and Surgical Quality, NorthShore University Health System, 2650 Ridge Avenue, 3507 Walgreen Buliding, Evanston, IL 60201, USA; ^5^Division of Thoracic Surgery, Emory University Healthcare, 1365 Clifton Rd NE, Atlanta, GA 30322, USA; ^6^Division of Thoracic Surgery, University of Minnesota, 420 Delaware Street SE, Mayo Mail Code 195, Minneapolis, MN 55455, USA; ^7^Division of Thoracic Surgery, Cedars Sinai Medical Center, 8635 West Third, Suite 675, Los Angeles, CA 90048, USA; ^8^Division of Cardiothoracic Surgery, Barnes-Jewish Hospital Plaza, Washington University in St. Louis, Queeny Tower, Suite 3108, St. Louis, MO 63110-1013, USA; ^9^Division of Minimally Invasive Thoracic Surgey, Brigham and Women's Hospital, Dana-Farber Cancer Institute, Harvard Medical School, 75 Francis Street, Boston, MA 02115, USA

## Abstract

This study examined the effect of surgeons' volume on outcomes in lung surgery: lobectomies and wedge resections. Additionally, the effect of video-assisted thoracoscopic surgery (VATS) on cost, utilization, and adverse events was analyzed. The Premier Hospital Database was the data source for this analysis. Eligible patients were those of any age undergoing lobectomy or wedge resection using VATS for cancer treatment. Volume was represented by the aggregate experience level of the surgeon in a six-month window before each surgery. A positive volume-outcome relationship was found with some notable features. The relationship is stronger for cost and utilization outcomes than for adverse events; for thoracic surgeons as opposed to other surgeons; for VATS lobectomies rather than VATS wedge resections. While there was a reduction in cost and resource utilization with greater experience in VATS, these outcomes were not associated with greater experience in open procedures.

## 1. Introduction

Lobectomies and wedge resections of the lung are performed using either open thoracotomy or minimally invasive techniques, particularly, video-assisted thoracoscopic surgery (VATS). The literature documents many purported benefits of VATS for major lung surgeries, such as smaller incisions, less pain, less blood loss, less respiratory compromise, faster recovery times translating into shortened hospital lengths of stay, and superior survival rates [1]. However, compared to open procedures, VATS has higher equipment costs, increased operating room times, and a learning curve for both surgeons and operating room personnel [2]. 

During the past three decades, a large body of empirical literature has established a positive relationship between provider volume and patient health outcomes across various medical and surgical procedures [3–10], with little attention paid to thoracic surgery. This is important, as the magnitude of the volume outcome effect was found to vary across health conditions and surgery procedures [8]. The reason that greater volume is associated with better throughput, clinical outcomes, and control over resources, is not well understood. This relationship may be the result of surgeons' “learning-by-doing” and/or the result of “selective referrals”, where physicians with better outcomes command a higher demand for their services [3].

To date, most of the work on volume outcome relations was conducted at the hospital level, as opposed to the surgeon level. In the case of lung surgery, patients who received open lobectomy and other resections at high-volume hospitals were less likely to experience postoperative complications and enjoyed better long-term and short-term survival rates [11–13]. A similar relationship between hospital volume and patient outcomes has been observed across patients receiving minimally invasive procedures; for example, minimally invasive endovascular interventions for patients with abdominal aortic aneurysms [14–16].

Recently, there is some evidence that the associations between hospital volume and operative mortality are mediated by surgeon volume [14, 17]. The volume of the surgeon was found to have a greater influence on patient outcomes than hospital volume [18]. This should come as no surprise, as hospital volume is the aggregate of all participating surgeons' volumes. Surgeons make preoperative and intraoperative decisions, affect case selection, and determine the appropriate surgical technique to be used. Studies of the relationship between surgeon volume and outcomes for cancer patients are mixed. A majority of cancer studies find that high-volume surgeons have a lower rate of operative mortality, with the strength of the relationship varying by condition and procedure [14, 19]. Conclusions may be obscured by heterogenous definitions of high-volume across studies and procedures [18].

Few studies have examined the relationship between surgeon volume and operative mortality for lobectomies and wedge resections [18, 20, 21]. In one such study, high volume surgeons were found to have less locoregional recurrence of cancer, but no differences were observed for mortality [20]. While thoracic surgeons were more likely to perform lobectomies and wedge resections using VATS, adjusting for surgeon and hospital volume, lung cancer patients treated by general thoracic surgeons had a lower probability of death than those treated by cardiothoracic surgeons or general surgeons [21]. A number of case studies based on either a single center or a single surgeon found greater experience with VATS to improve such patient outcomes as blood loss, recurrence, operation time, surgeon-related thoracotomy conversions, and readmissions [22–24].

Understanding volume-outcome relationships is of considerable practical importance because it quantifies the effects of experience on clinical outcomes. However, experience must be relevant to performance. Even though surgeons often use different techniques (e.g., open procedure versus VATS), studies have not accounted for technique-specific experience in calculating volume. To our knowledge, this is the first study to accumulate experience with VATS separately from experience with open procedure, as the two techniques command different surgical skills. Information regarding skill development through practice is an important factor that may affect patient decisions of where to seek treatment and provider decisions about where to refer their patients. Furthermore, transitioning from open to VATS procedures is not trivial, hence it is important to study the degree of transferability of experience across the two procedures [25].

Volume-outcome studies of cancer patients have reported mortality, inpatient length-of-stay, readmissions, and several specific clinical indicators, such as blood loss and perioperative complications [26, 27]. However, greater experience can manifest itself in additional ways. Recent studies documented variations among physicians in their ability to shorten the length-of-stay for their patients, reduce resource utilization, improve quality, and reduce the likelihood of hospital-borne infections.

This current work aims to quantify the impact of a surgeon's volume on outcomes in lung surgery, adjusted for other potential explanatory variables. We studied performance on lobectomies and wedge resections separately and accounted for the experience of surgeons as represented by six-month case volumes using both VATS and open techniques. Also, we analyzed the effect of this technique-specific experience on inpatient costs, length of surgery, length of stay, as well as the likelihood and number of adverse surgical events.

## 2. Materials and Methods

A protocol describing the analysis objectives, criteria for patient selection, data elements of interest, and statistical methods was submitted to the New England Institutional Review Board (NEIRB), and exemption was obtained.The study was funded by Ethicon Endo-Surgery Inc. (Cincinnati, Ohio, USA).

### 2.1. Data Source

This study utilizes the Premier Hospital Database, which contains clinical and utilization information on patients receiving care in over 600 USA hospitals and ambulatory surgery centers across the nation. The database contains complete patient billing, hospital cost, and coding histories from more than 25 million inpatient discharges and 175 million hospital outpatient visits. Since VATS is a new technology, the analyzable dataset was restricted to procedures occurring in 2007-2008. Only data that were anonymized with regard to patient identifiers were used.

### 2.2. Patients and Procedures

Eligible patients were those of any age undergoing VATS lobectomy or wedge resection for cancer. International Classification of Diseases, 9th Revision (ICD-9) diagnosis codes and procedure codes for identifying lobectomy and wedge resection procedures, cancer diagnoses, comorbid conditions, and all adverse events are listed in Tables 7–10.

### 2.3. Volume Outcome Variable

The volume measure typically used in previous research utilized subsequent volume to predict outcomes. For example, many studies defined physician volume as the number of surgeries done over a specific time period and used that measure to predict outcomes of each surgery performed within that same time period [8, 9, 12, 14, 28]. As a result, experience not yet acquired was used to describe current performance, which could potentially overestimate the influence of volume on surgeon outcomes. 

For each outcome-surgeon combination, our measure of volume represented the aggregate experience level of the surgeon. Volume-accumulated experience over running six-month windows involved recording surgeons' volume at a given date as the number of procedures accumulated during the prior six months. This measure is more precise than fixed calendar periods and was used extensively in the literature, as it responds instantaneously to any changes in the surgeon's recent experience profile. Experience accumulation with moving, rather than fixed, windows can be viewed as smoothing the calendar step function and alleviating the imprecision that increases for observations occurring toward the end of the observation period [29].

### 2.4. Statistical Analyses

Initial counts, percentages, means, and standard deviations for patient demographics, comorbid conditions, hospital characteristics, as well as safety utilization and cost outcomes were summarized separately for VATS lobectomy versus VATS wedge resection and separately for thoracic surgeons versus all surgeons using descriptive statistics. Type of surgeon (thoracic versus general) was identified via physician identification codes provided in the database.

The safety outcomes of interest were pertinent adverse events occurring during or up to 30–60 days after surgery. A dichotomous variable was used indicating the existence of an adverse event as well as a continuous variable tallying the number of adverse events. Utilization outcomes were surgery duration (hours) and hospital length of stay (days). Cost outcomes were total hospital costs per patient, both fixed and variable. Since we only studied VATS procedures, we did not include costs for initial acquisition of the VATS equipment.

In addition, descriptive statistics for the volume explanatory variables are presented. The key explanatory variable was each surgeon's volume for lobectomy and wedge resection using VATS or open thoracotomy techniques. This measure of volume corresponded to the aggregate experience level of the surgeon over running six-month windows. Experience with open thoracotomy procedures may or may not contribute to performance with VATS, but it is certainly expected that experience specific to VATS will be the most relevant in explaining outcomes for patients treated with VATS.

Multivariable logistic regression analyses were estimated for the adverse event binary outcome: the presence or absence of specific individual events. Ordinary least squares (OLS) regression was used for all other continuous outcomes such as hospital costs, surgery time, length of stay, and number of adverse events. For all models, in addition to the volume measures, the following explanatory variables were included: age, gender, race, marital status, insurance type, diagnosis (metastasis versus primary cancer), comorbid conditions (e.g., diabetes), All Patient Refined-Diagnosis-Related Groups (APR-DRGs) severity index (an index of comorbidity unique to the Premier database that reflects preoperative severity level), census region of hospital, rural versus urban hospitals, teaching versus nonteaching hospitals, and number of hospital beds. 

Using these explanatory variables, multivariable models were estimated to isolate the effects of a surgeon's VATS volume on adverse events, hospital costs, surgery time, and length of stay. Because the cost and utilization variables were right skewed, they were converted to natural logarithms to normalize their distributions, although the results were not sensitive to this transformation. Missing data or values of zero were not included in the OLS regression models. Weights provided in the Premier database were used to transform the results in a manner that permitted generalizability to the USA population. All analyses were performed using Stata Version 10 (StataCorp LP, College Station, Texas, USA).

## 3. Results

**Figure 1:**
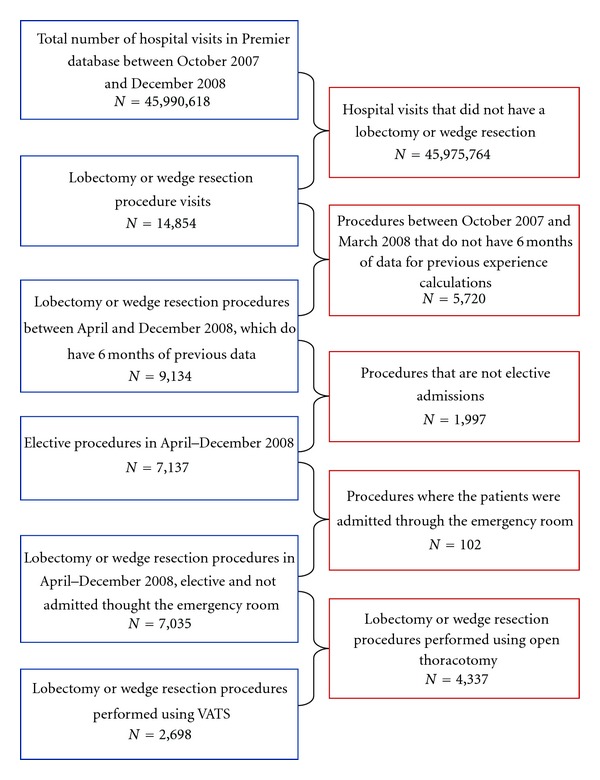
Attrition diagram. Thoracotomy: open versus VATS.

Of 7,137 patients in the database with elective, inpatient resections for lung cancer, a total of 2,698 patients underwent lobectomy (*n* = 716) or wedge resection (*n* = 1982) using VATS. More than 70% of these procedures were performed by thoracic surgeons (*n* = 1,896). A patient attrition diagram is shown in Figure 1. Characteristics of eligible patients are summarized in Table 1. There were slightly more females than males in all four samples, and most patients in all samples were over 60 years of age and covered by Medicare. Most patients were Caucasian, with primary (as opposed to metastatic) neoplasm of the lung and only minimal to moderate illness severity level, as measured by the APR-DRG severity index. As expected, the severity index for patients undergoing lobectomy was higher than for patients undergoing wedge resection. Patient characteristics within procedure (lobectomy versus wedge resection) were similar across the thoracic surgeons sample and the all surgeons sample.

The distribution of specific patient comorbidities is shown in Table 2. The most frequent comorbidities reported were chronic obstructive pulmonary disease (COPD), diabetes mellitus, and heart disease. The distribution of these conditions is similar across all samples.

A total of 237 hospitals contributed data on VATS lobectomies and wedge resections. Patient-weighted hospital characteristics for the four samples are reported in Table 3. Compared with patients undergoing VATS wedge resection, patients undergoing VATS lobectomy were more likely to receive the procedure in a teaching hospital (63% versus 57%) and in a hospital with over 600 beds (46% versus 38%). All samples exhibit similar demographic distributions.

Average hospital costs, surgery time, length of hospital stay, the likelihood, and number of adverse events, as well as the surgeons' volume measures for each sample were examined prior to multivariable modeling. The data suggest that, on average, VATS lobectomies cost hospitals more than VATS wedge resections ($19,697 versus $13,058) are associated with both longer surgery time (four hours versus 2.5 hours) and longer lengths of hospital stay (5.7 days versus 3.9 days). Furthermore, patients undergoing lobectomy had a higher likelihood of experiencing an adverse event compared to patients undergoing wedge resection (0.57 versus 0.43) and had a higher number of adverse events on average (1.13 events versus 0.72 events).

This study tracks 575 surgeons performing lobectomies or wedge resections using VATS (366 of whom were thoracic surgeons). Patients treated by thoracic surgeons using VATS lobectomy had lower inpatient costs and shorter length of stay compared with patients seen by general and other surgeons. While these effects were statistically significant at the 1% level, they were evidently small. No other statistically meaningful differences between thoracic and other surgeons were found for patients treated using VATS wedge resection or for other outcomes (i.e., length of surgery, likelihood of adverse event, and number of adverse events).

Surgeons' six months experience with VATS varies by sample (Table 4). The most experienced surgeons, on average, are found in the sample of thoracic surgeons performing VATS lobectomies, 31.6 procedures. This average decreases to 22.3 procedures when considering all surgeons performing VATS wedge resections. Six months experience, for these surgeons, with open lobectomies and open wedge resection was lower, 5.4 procedures and 3.9 procedures, respectively, for the entire sample.

### 3.1. Multivariable Findings

Given the possibility of confounders in these group comparisons of outcomes, we performed multivariable regression analyses, adjusting for a number of potential confounders, including patient demographics, metastatic versus primary cancer, comorbid conditions, APR-DRG severity index, and hospital characteristics. The results of these adjusted analyses of costs, surgery time, length of stay, likelihood of adverse event, and the number of adverse events are shown in Table 5. For ease of interpretation, we report the estimated marginal effects for each one of the 40 models presented in Table 5. The reported marginal effects measure the expected instantaneous change in each one of our five-outcome variables as a function of a change in surgeons' VATS volume, while keeping all the other covariates constant. Note that, for each outcome of interest, we compared the estimated marginal effects obtained from an unadjusted analysis with the estimated marginal effects from the multivariable analysis described above. (Note: only adjusted findings are reported in Table 5).

In the unadjusted analysis for the all surgeons lobectomy sample, doubling the average surgeon's volume was associated with a 10% reduction in inpatient cost ($2,029), a 5% reduction in surgery time (13 minutes), and a 15% reduction in length-of-stay (approximately one day). The effect of experience on the likelihood of an adverse event, while statistically significant, was small in magnitude. Increased surgeons' experience was associated with a reduction of one adverse event in one of every five patients. Even after adjusting for the variables detailed in Tables 1 through 3, all the findings above persist. 

The first and second columns of Table 5 reports the analysis for lobectomies for all surgeons and then surgeries performed exclusively by thoracic surgeons. For the most part, the volume-outcome relationship for thoracic surgeons is stronger. Doubling of the thoracic surgeons experience was associated with a 13% reduction in inpatient cost ($2,409) and a 7% reduction in surgery time (18 minutes). All other results were similar to the ones obtained for all surgeons.

The second and third columns of Table 5 repeat the analysis for patients undergoing VATS wedge resection. Here, for most outcomes and specifications, the volume-outcome relationship appears much weaker. Doubling of the surgeon's experience was associated with a 3% reduction in inpatient cost ($389), a 2% reduction in surgery time (3 minutes), and an 8% reduction in hospital length of stay (a third of a day). The results were similar when considering the most saturated model and when limiting the sample to procedures performed solely by thoracic surgeons. The only exception was the reduction in cost for the thoracic surgeon sample, which was 5% ($659).

Table 6 reports results from models similar to those reported in Table 5, and includes two additional variables: the surgeon's six-months experience with open lobectomies and the surgeon's six-months experience with open wedge resections. The two additional volume measures allow for assessing the contribution of competing sources of learning. For example, for the VATS lobectomy sample, one may argue that any experience with lobectomy (open or VATS) may be an important contributor for performance. This is tested directly in Table 6. Overall we find the volume-outcome relationship for experience with VATS to be similar in sign, magnitude, and statistical significance to those described in Table 5. Experience with open lobectomy did not have an effect on outcomes for patients treated with VATS lobectomy, with the exception of the number of adverse events, where greater experience with open lobectomy was associated with a small reduction in the number of adverse events for VATS lobectomy. Similarly, experience with open wedge resection was associated with a reduction in inpatient cost and length of stay beyond the reductions associated with greater experience with VATS.

## 4. Discussion

An important strength of the Premier database is that it provides very large numbers of patients, surgeons, and procedures on a nationwide scale. Obtaining this extremely large sample size from a practical setting allows researchers to better understand processes such as the relationship between surgeons' volume and outcomes. In turn, this analysis provides hospitals, patients, and surgeons with a quantifiable measure of the benefits of surgeons' volume on outcomes in lung surgery. The sample size and large number of elements in the Premier database allows for analyzing the effect of experience with VATS on inpatient costs, length of surgery, length of stay, as well as the likelihood and number of adverse surgical events.

In this retrospective analysis, we find evidence of volume-outcome relationship. The relationship is stronger (1) for cost and utilization outcomes as opposed to adverse events, (2) for thoracic surgeons rather than other surgeons, and (3) for VATS lobectomy procedures more than for VATS wedge resection procedures. Finally, we find that while there was a reduction in cost and resource utilization associated with greater experience with VATS, these outcomes were not strongly linked with greater experience with open procedures. Thus, by and large, performance with VATS is associated primarily with experience with VATS.

The choice between VATS and open lobectomy has implications for the surgeon's learning profile, as the reduction in cost and resource utilization associated with greater experience with VATS were much larger than those associated with greater experience with open procedures. This finding reinforces the need for surgeons' specialization and centralization of delivery for VATS.

There were certain limitations of this study. This is a retrospective analysis from a transactional database (Premier) and not a prospective analysis where randomization and more detailed information about patients and procedures could be collected. For instance, it would have been of interest to examine the influence of additional patient characteristics, such as weight or BMI, and more procedure-related details. Nevertheless, we include numerous controls in our analysis, particularly, controls for patient characteristics [30] and hospital characteristics [12].

Another limitation, and a topic that can be the focus of future research, is the lack of information on surgeons' characteristics. In particular, data associated with surgeons' characteristics (e.g., years in practice, graduate of which medical school, completion of fellowship, etc.) would be of interest. This information may be important as surgeons do not randomly adopt VATS, and the results may therefore be biased if the most able surgeons are also the ones who adopt and utilize VATS extensively.

## 5. Conclusions

Our analysis of a large, nationally representative hospital database revealed three key findings: (1) there is a reduction in cost and resource utilization associated with greater experience with VATS, especially for VATS lobectomy for lung cancer; (2) thoracic surgeons have better VATS outcomes than non-thoracic surgeons; (3) greater experience with open procedures does not correlate with better VATS outcomes. These findings have implications for the organization of health care delivery of both minimally invasive and open procedures.

## Figures and Tables

**Table 1:** Patient characteristics.

Procedure*	VATS lobectomy		VATS wedge resection	
****	All surgeons (including thoracic)	Thoracic surgeons (only)	All surgeons (including thoracic)	Thoracic surgeons (only)
Total *N*	716	546	1,982	1,350
(% of total *N* = 2,698)	(26.54%)	(20.24%)	(73.46%)	(50.04%)
Age average (SD)	66.68 (11.27)	66.51 (10.96)	61.09 (15.39)	61.37 (14.57)
<40	0.01	0.01	0.10	0.09
41–50	0.07	0.08	0.12	0.12
51–60	0.20	0.20	0.20	0.21
61–70	0.32	0.33	0.29	0.29
71–80	0.30	0.28	0.24	0.24
>80	0.10	0.10	0.06	0.06
Race				
Caucasian	0.79	0.84	0.74	0.79
African American	0.07	0.07	0.08	0.09
Other	0.13	0.10	0.18	0.13
Gender				
Female	0.55	0.55	0.53	0.53
Male	0.45	0.45	0.47	0.47
Marital status				
Married	0.59	0.62	0.54	0.58
Unmarried	0.41	0.38	0.46	0.42
Insurance type				
Commercial	0.07	0.07	0.07	0.07
Medicare	0.60	0.60	0.49	0.49
Medicaid	0.03	0.02	0.05	0.05
Managed care	0.28	0.30	0.35	0.35
Other	0.03	0.02	0.04	0.03
Malignancy indication**				
Primary neoplasm of the lung	0.96	0.95	0.86	0.85
Metastases from other primary malignancy	0.04	0.05	0.14	0.15
Illness severity level				
APR-DRG Severity Level (1, 2)	0.78	0.79	0.87	0.86
APR-DRG Severity Level (3, 4)	0.22	0.21	0.13	0.14

^∗^All procedures are inpatient. CPT and ICD codes for resections in Table 7.

**ICD codes for lung cancer in Table 8.

**Table 2:** Comorbid conditions*, **.

	VATS lobectomy		VATS wedge resection	
	All surgeons (including thoracic)	Thoracic surgeons (only)	All surgeons (including thoracic)	Thoracic surgeons (only)
Total *N* (2,698)	716	546	1,982	1,350
Myocardial infarction, acute or old	0.11	0.12	0.08	0.09
Congestive heart failure	0.07	0.07	0.07	0.08
Other chronic or unspecified heart failure	0.02	0.02	0.02	0.03
Peripheral vascular disease	0.10	0.10	0.08	0.08
Dementia	0.03	0.03	0.02	0.01
Chronic pulmonary disease	0.50	0.50	0.47	0.49
Connective tissue disease	0.03	0.03	0.05	0.05
Liver disease	0.05	0.05	0.06	0.06
Chronic viral hepatitis	0.01	0.01	0.01	0.01
Renal insufficiency, chronic	0.04	0.04	0.05	0.05
Diabetes mellitus	0.19	0.18	0.19	0.20

*Proportions of comorbid conditions existing for patients any time during or before procedure stay in Premier database (beginning in 2000).

**ICD codes for these variables are found in Table 10.

**Table 3:** Hospital characteristics.

	VATS lobectomy		VATS wedge resection	
	All surgeons (including thoracic)	Thoracic surgeons (only)	All surgeons (including thoracic)	Thoracic surgeons (only)
Total *N* (2,698)	716	546	1,982	1,350
Census region				
Northeast	0.22	0.22	0.21	0.17
Midwest	0.12	0.11	0.24	0.23
South	0.51	0.51	0.41	0.45
West	0.15	0.16	0.14	0.15
Location				
Urban	0.96	0.96	0.95	0.96
Nonurban	0.04	0.04	0.05	0.04
Type				
Teaching	0.63	0.62	0.57	0.57
Nonteaching	0.37	0.38	0.43	0.43
Bed count				
<200	0.05	0.04	0.06	0.05
200–400	0.20	0.21	0.26	0.25
400–600	0.29	0.31	0.30	0.35
>600	0.46	0.44	0.38	0.35

**Table 4:** Volume and outcomes measures*.

	VATS lobectomy		VATS wedge resection	
	All surgeons (including thoracic)	Thoracic surgeons (only)	All surgeons (including thoracic)	Thoracic surgeons (only)
Total *N* (2,698)	716	546	1,982	1,350
Inpatient costs (dollars)	19,697	19,271	13,058	13,127
	[10,670]	[10,934]	[8,669]	[9,157]
Length of surgery (hours)	4.079	4.008	2.537	2.557
	[1.477]	[1.439]	[1.079]	[1.098]
Length of stay (days)	5.753	5.676	3.944	3.952
	[4.122]	[4.314]	[3.384]	[3.426]
Likelihood of adverse event	0.571	0.557	0.435	0.436
	[0.495]	[0.497]	[0.496]	[0.496]
Number of adverse events	1.126	1.092	0.722	0.740
	[1.361]	[1.347]	[1.062]	[1.094]
VATS six-month volume	28.42	31.64	22.30	24.59
	[30.80]	[33.57]	[27.11]	[30.56]
Open lobectomy six-month volume	5.27	5.46	5.52	5.38
	[4.54]	[4.51]	[5.48]	[4.85]
Open wedge Res six-month volume	3.66	3.73	4.02	3.97
	[3.11]	[2.89]	[3.75]	[3.43]

^∗^ Standard deviations are reported in brackets.

^∗∗^ ICD codes for these variables are found in Table 10.

**Table 5:** Multivariable results for cost, utilization, and adverse events.

	VATS lobectomy		VATS wedge resection	
	All surgeons (including thoracic)	Thoracic surgeons (only)	All surgeons (including thoracic)	Thoracic surgeons (only)
Total *N* (2,698)	716	546	1,982	1,350
Inpatient costs (dollars)				
Regression coefficient	−0.066***	−0.098***	−0.0436***	−0.0468***
	[0.0158]	[0.0170]	[0.00904]	[0.0104]
Length of surgery (hours)				
Regression coefficient	−0.045***	−0.074***	−0.0475***	−0.0317***
	[0.0135]	[0.0149]	[0.0077]	[0.0084]
Length of stay (days)				
Regression coefficient	−0.096***	−0.117***	−0.0778***	−0.0665***
	[0.0207]	[0.0237]	[0.0129]	[0.0141]
Likelihood of adverse events				
Regression coefficient	−0.002*	−0.002***	−0.0005	−0.00098
	[0.0009]	[0.0010]	[0.0006]	[0.0007]
Number of adverse events				
Regression coefficient	−0.083***	−0.142***	−0.0119	−0.0273
	[0.0396]	[0.0493]	[0.0269]	[0.0292]

Estimated marginal effects are reported, standard deviations are reported in brackets * and *** indicate significance at the 10% and 1% levels.

**Table 6:** Multivariable results for cost, utilization, and adverse events (including non-VATS volume).

	Lobectomy for all surgeons			Lobectomy for Thoracic Surgeons only			Wedge Resection for All Surgeons			Wedge Resection for Thoracic Surgeons		
											only	
	VATS surgeon volume	Open lobectomy volume	Open wedge volume	VATS surgeon volume	Open lobectomy volume	Open wedge volume	VATS surgeon volume	Open lobectomy volume	Open wedge volume	VATS surgeon volume	Open lobectomy volume	Open wedge volume
Cost (dollars)												
Regression coefficient (marginal effects)	−0.062***	−0.014	0.044	−0.092***	0.006	−0.058***	−0.034***	−0.037***	−0.0043	−0.0474***	−0.0682***	−0.0077
Standard deviation	[0.0162]	[0.026]	[0.0287]	[0.0172]	[0.0277]	[0.0274]	[0.0093]	[0.0136]	[0.0163]	[0.0103]	[0.0149]	[0.0181]
Length of Surgery (Hours)												
Regression coefficient (marginal effects)	−0.043***	−0.003	−0.0195	−0.074***	−0.002	−0.0100	−0.032***	−0.083***	−0.017	−0.0339***	−0.089***	−0.022
Standard deviation	[0.0137]	[0.0239]	[0.0262]	[0.0152]	[0.0295]	[0.0277]	[0.0076]	[0.0100]	[0.0118]	[0.0084]	[0.0124]	[0.0251]
Length of Stay (Days)												
Regression coefficient (marginal effects)	−0.087***	0.022	−0.062*	−0.104***	0.048	−0.079***	−0.063***	−0.062***	−0.0005	−0.066***	−0.069***	0.0036
Standard deviation	[0.0162]	[0.027]	[0.0325]	[0.0234]	[0.0318]	[0.0361]	[0.0128]	[0.0214]	[0.0233]	[0.0141]	[0.0236]	[0.0123]
Likelihood of Adverse Events												
Regression coefficient (marginal effects)	−0.002*	−0.009	−0.005	−0.002***	−0.012	−0.009	−0.0002	−0.0035	−0.0009	−0.00085	−0.0033	−0.00595
Standard deviation	[0.0009]	[0.008]	[0.010]	[0.0010]	[0.011]	[0.011]	[0.0006]	[0.0034]	[0.0058]	[0.0007]	[0.0046]	[0.0061]
Number of Adverse Events												
Regression coefficient (marginal effects)	−0.005***	−0.032***	0.013	−0.163***	−0.180***	0.033	0.0022	−0.1005***	0.0398	−0.0289	−0.0998***	−0.0039
Standard deviation	[0.0019]	[0.0143]	[0.0208]	[0.0498]	[0.0732]	[0.0729]	[0.0278]	[0.0389]	[0.0455]	[0.0292]	[0.0428]	[0.0427]

Estimated marginal effects are reported, standard deviations are reported in brackets, * and *** indicate significance at the 10% and 1% levels.

**Table 7:** 

Pulmonary lobectomy CPT codes and ICD-9 codes sets	
Open procedures	
CPT 32480	Removal of lung, other than total pneumonectomy; single lobe (lobectomy)
ICD 32.49	Other lobectomy of lung
VATS procedures (i.e., via thoracoscopy)	
CPT 32663	Thoracoscopy, surgical; with lobectomy, total or segmental
ICD 32.41	Thoracoscopic lobectomy of lung

Wedge resection CPT codes and ICD-9 codes sets	
Open procedures	
CPT 32484	Removal of lung, other than total pneumonectomy; single segment (segmentectomy)
CPT 32500	Wedge resection
ICD 32.39*	Other and unspecified segmental resection of lung
ICD 32.29	Other local excision or destruction of lesion or tissue of lung (used for wedge resection)
VATS procedures (i.e., via thoracoscopy)	
CPT 32657	Thoracoscopy, surgical; with wedge resection of lung, single or multiple
ICD 32.30*	Thoracoscopic segmental resection of lung
ICD 32.20	Thoracoscopic excision of lesion or tissue of lung (used for thoracoscopic wedge resection)

^∗^Codes 32.30 and 32.39 became effective on October 1, 2007. Prior to that date, the codes were simply 32.3 which did not differentiate between open and thoracoscopic excisions. Due to this lack of information in the ICD codes, data for this project was limited to discharges on or after October 1, 2007.

**Table 8:** ICD-9 codes for index diagnosis.

Indications for surgery	
Malignant	
Primary neoplasm of the lung	162.x, 209.21*
Metastatic site	197.0

^∗^ICD code 209.21 (malignant carcinoid tumor of the lung) came into existence on October 1, 2008. Prior to October 1, 2008, this type of lung cancer was coded together with 162.x.

**Table 9:** Postoperative procedure-specific complications.

Postoperative procedure-specific complications*		Postoperative code
Pulmonary		
Acute respiratory failure	518.81, 518.84, 518.5	997.39
Spontaneous tension pneumothorax	512.0	997.39
Atelectasis/pulmonary collapse	518.0	997.39
Empyema	510.9	998.59
Bronchopleural fistula	510.0	998.59
Air leak and other pneumothorax	512.1, 512.8	
Chylothorax	457.8	
Pneumonia	480.x to 486, 507.0	997.39
Other pulmonary infections and inflammation	487.0, 490, 491.21–491.22, 511.0–511.1, 511.89, 511.9, 513.x, 519.01	997.39
Cardiac		
Arrhythmia	427.xx	997.1
Acute myocardial infarction	410.xx	997.1
Acute heart failure/pulmonary edema	428.1, 428.21, 428.23, 428.31, 428.33, 428.41, 428.43, 514, 518.4	997.1
Vascular/thromboembolic		
Acute pulmonary embolism/infarction	415.1x	
Acute deep venous thrombosis of extremities	453.4x, 453.8, 453.9	
Neurological		
Acute cerebrovascular accident (stroke)	433.x1, 434.x1, (997.02)	997.02
Transient cerebral ischemia/attack (TIA)	435.x, 437.1	997.09
Intracranial hemorrhage (includes hemorrhagic stroke)	430–432.x	997.02
Wound complications		
Dehiscence	998.30, 998.31, 998.32, 998.3	
Hematoma/seroma complicating a procedure	998.12–998.13, 998.51	
Cellulitis	998.59 plus 682.2	
Other postoperative infection, including other (noncellulitis) wound infection	998.59 when 510.9, 510.0, 038.xx, 790.7, 995.9x, 682.2 are NOT also present	
Other		
Perforations organ or vessels	998.2	
In-hospital deaths	Obtained from Premier variable	
Sepsis	038.xx, 790.7, 995.9x	998.59
Other postoperative complications	997.xx EXCEPT 997.02, 998.0, 998.11, 998.33, 998.4, 998.6, 998.7, 998.8*x*, and998.9	
Conversion from /VATS to OPEN	V64.42	

^∗^All procedures are inpatient.

**Table 10:** Comorbid Conditions.

Comorbid conditions (existing for patient any time during or before procedure stay in Premier data)	
Myocardial infarction, acute or old	410.xx, 412
Congestive heart failure	428.0
Other chronic or unspecified heart failure	428.20, 428.22, 428.30, 428.32, 428.40, 428.42, 428.9
Peripheral vascular disease	440.xx, 443.8x, 443.9
Dementia	290.xx, 294.xx, 331.0, 331.11, 331.19, 331.2, 331.7, 331.82
Chronic pulmonary disease	490.xx–494.xx, 495.x, 496, 500–505
Connective tissue disease	710.xx, 714.xx
Liver disease	571.x, 572.*x*, 573.xx
Chronic viral hepatitis	070.22–070.23, 070.32–070.33, 070.44, 070.54
Renal insufficiency, chronic	585.xx
Diabetes mellitus	249.xx, 250.xx
